# Association between sleep duration and dental caries in a nationally representative U.S. population

**DOI:** 10.1186/s12903-023-03147-z

**Published:** 2023-07-18

**Authors:** Abdullah Alawady, Asma Alharbi, Hajar Alharbi, Sarah Almesbah, Noor Alshammari, Ahmad Alkandari, Hesham Alhazmi, Hend Alqaderi

**Affiliations:** 1 Ministry of Health, Sulaibikhat, Kuwait; 2 Department of Preventive Dentistry, Faculty of Dentistry, Division of Pediatric Dentistry, Umm Al-Qura University, Makkah, Saudi Arabia; 3 Department of Oral Health Policy and Epidemiology, Harvard School of Dental Medicine, Boston, United States of America; 4 Dasman Diabetes Institute, Dasman, 1180 Kuwait

**Keywords:** Sleep duration, Dental caries, Decay, Diet, Saliva, NHANES

## Abstract

**Background:**

Dental caries is considered one of the most prevalent chronic diseases worldwide despite all dental public health efforts. Short sleep duration has been established as a risk factor for several medical conditions. In this study, we aimed to examine the relationship between sleep duration and dental caries.

**Methods:**

Data were collected from the 2017–2018 cycle of the National Health and Nutrition Examination Survey, a nationally representative health survey conducted in the United States. Participants who completed sleep questionnaires were examined by dentists using standardized clinical criteria. Analysis was limited to Individuals aged ≥ 16 years with complete clinical oral examination data and who completed the sleep questionnaire (N = 5,205). The data were weighted to provide a national estimate, and multiple potential covariates were included in the analysis to account for the complex sample design. The main outcomes of the study were untreated dental caries and dental caries experience. The main predictor variables were average sleep hours/night and a binary variable with 7 h/night as a cut off. Multiple weighted Poisson and logistic regression analyses were conducted to test the hypothesis that people with short sleep duration are more likely to exhibit dental caries.

**Results:**

This study showed a statistically significant negative relationship between sleep duration and dental caries amongst all weighted adjusted analyses conducted. For a one hour increase in average sleep hours, the Adjusted Odds Ratio (AOR) of having a dental caries experience might decrease by 0.86 (AOR = 0.86, 95% CI = 0.75–0.98, P < 0.05). Individuals who reported an average sleep of ≥ 7 h were less likely to have a dental caries experience compared to individuals who reported an average sleep of < 7 h (AOR = 0.52, 95% CI = 0.33–0.82, P < 0.05). For a one hour increase in average sleep hours, the Adjusted Mean Ratio (AMR) of having a dental caries experience might decrease by 0.97 (AMR = 0.97, 95% CI = 0.96–0.99, P < 0.05), and was lower for those who reported sleeping ≥ 7 h/night than individuals who reported sleeping < 7 h/night (AMR = 0.92, 95% CI = 0.87–0.99, P < 0.05).

**Conclusion:**

Findings of this cross-sectional representative study of the U.S. population revealed a statistically significant negative association between sleep duration and dental caries. In this study, individuals who slept < 7 h/night were more likely to exhibit dental caries.

## Background

Dental caries is a multifactorial, preventable, chronic, and biofilm mediated disease. It is one of the most prevalent chronic diseases worldwide affecting approximately half the global population. Symptoms of dental caries include pain, infection, and compromised esthetics negatively affecting an individual’s quality of life through its impact on oral health [1].

Multiple risk factors have been identified to be associated with dental caries in the literature including physical, biological, environmental, behavioral, and lifestyle-related factors [2].

Over the years, numerous studies have accumulated a robust body of evidence demonstrating that individuals with low socioeconomic status and limited access to dental care are at a higher risk of developing dental caries [3]. Additionally, the commercial sector, especially food and beverage companies, plays a crucial role in promoting unhealthy diets that contribute to the development of dental caries [3]. This influence is particularly significant among individuals with lower levels of education, as it can impact their oral health behaviors and outcomes.

Currently, most interventions that target dental caries focus on diet and tooth brushing, the most evident risk factors for dental caries. Despite all efforts, the prevalence of dental caries is still considered high and is increasing in low- and mid-income countries [3]. Thus, it is worth exploring other risk factors of dental caries that might mediate and explain the biological mechanism of dental caries development.

Sleep is a multifaceted and indispensable biological process that is necessary on a daily basis for all individuals, regardless of age, gender or cultural background [4]. Studies have identified poor sleep schedule to be a known risk factor for multiple adverse health outcomes, such as diabetes, cardiovascular disease, obesity, and several inflammatory disorders [5–7].

Researchers have deemed sleep to be a vital component of the immune system. Sleep deprivation can lead to a diminished immune response, thereby increasing the likelihood of infection [8]. Additionally, it has been observed that alterations in cortisol, catecholamines, prolactin, growth hormone and melatonin, which are regulated by sleep, frequently have an effect on immune functions and fighting pathogens [9].

Furthermore, a systematic review and meta-analysis investigating the relationship between sleep and inflammation has shown that sleep deprivation is closely linked to high levels of inflammation markers [10]. This increase in inflammation markers has the potential to exacerbate the risk of oral infection and contribute to the development of dental caries.

Sleep imbalance has been shown to disrupt the body’s natural hormonal balance, which may in turn adversely affect the immune system leading to susceptibility to infections [11]. Recent evidence has shown a link between short sleep duration and altered specific hormones that influence appetite leading to an increase in appetite [12]. Decreased sleep duration is also associated with an increased frequency of snacking and hence increased risk of developing dental caries due to the carbohydrate-rich snacks providing sustenance for the cariogenic biofilm. Furthermore, studies have linked sleep deprivation with decreased salivary flow and a disruption in the natural salivary proteome [13].

There is scant evidence in the literature about the relationship between sleep duration and dental caries in adults. Currently, the published studies are focused on pediatric populations and deciduous teeth [14, 15]. Our study addresses this gap in the literature as understanding this relationship may help us manage and prevent dental caries in the future more effectively.

The aim of this study was to determine the association between sleep duration and dental caries using a representative sample of the United States population. We hypothesize that short sleep duration would be associated with an increased risk of dental caries.

## Materials and methods

### Study design and population

Data were extracted from the publicly available National Health and Nutritional Examination Survey (NHANES) database.

NHANES is conducted by the US Center for Disease Control and Prevention (CDC). It is a cross-sectional survey of non-institutionalized civilians selected to represent the US population of all ages. The data is collected annually and publicly released in two-year cycles since 1999. The NHANES includes detailed demographic and health questionnaires, laboratory assays, and clinical examination measures of health outcomes. All individuals participating in the survey were given written consent forms.

NHANES uses a complex, multistage sampling strategy to select a representative sample of the US population. The strategy involves selecting a random sample of counties and then selecting a random sample of households within those counties. Within each household, eligible individuals are randomly selected to participate in the survey. The sampling is designed to oversample certain subpopulations, such as minorities and low-income individuals, to ensure adequate representation [16].

**Fig. 1:**
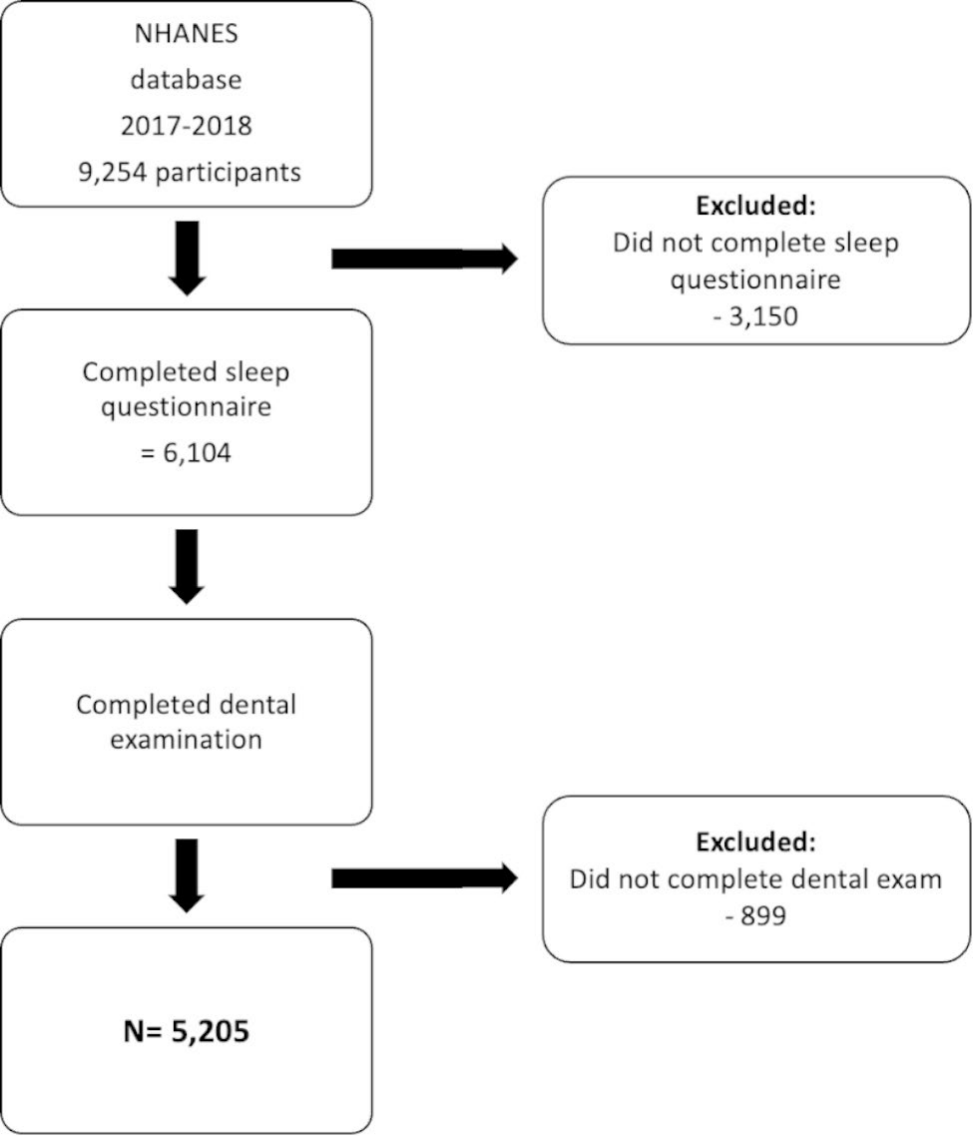
Flowchart of the screening process for the selection of eligible participants in this study

The dataset from 2017 to 2018 was selected as it was the most recent complete data set with all the required variables. More recent datasets were excluded as they were incomplete due to operational disruptions caused by the COVID-19 pandemic. Participants were excluded if they did not complete the sleep questionnaire or if their dental examination was incomplete. Since the sleep questionnaire was administered only to individuals aged 16 and over, all individuals aged < 16 were excluded from the study. The total sample size included in this cross-sectional study was 5,205 (Fig. 1).

The data extraction was conducted in compliance with the Data Use Restrictions for data collected by the National Center for Health Statistics, Centers for Disease Control and Prevention.

### Outcome variable(s)

Dental surveys and examinations were conducted by calibrated dental providers on Mobile Examination Centers. The caries scoring criteria and calibration details are described in depth in the NHANES plan and operations manual [16].

In brief, dental professionals by the NHANES conducted and collected dental exam data. The main outcomes in this study were: (1) presence of untreated dental caries. This variable was defined as the presence of at least one tooth with untreated dental caries, and (2) dental caries experience. This variable was defined as the presence of at least one decayed, missing, or filled tooth due to caries.

The NHANES variable “0HX05CTC” was coded for each tooth, excluding wisdom teeth, and represents coronal caries. For the “untreated dental caries” variable, we included the codes: J and Z. For the “dental caries experience” variable, we included the codes: E, F, J, P, R, T, and Z. The key for the codes is available elsewhere [16].

Finally, we created the “untreated dental caries” and “dental caries experience” variables by merging the variable representing the teeth present/absent with the variable representing the coronal caries status for each outcome variable as mentioned above.

The outcome variables were then dichotomized into binary variables. The “untreated dental caries” binary variable represents the presence or absence of untreated dental caries, while the binary “dental caries experience” represents the presence or absence of decayed, missing, or filled teeth.

### Predictor variables

The main predictor variable was sleep duration. This variable was coded using questions derived from the Mobile Examination Center (MEC) Questionnaire. The following sleep habit questions were selected (1) “Number of hours usually sleep on weekdays or workdays?” (2) “Number of hours usually sleep on weekends or non-workdays?”.

Using this data, the average sleep hours per night was calculated using the following formula: [(sleep duration weekday × 5) + (sleep duration weekend × 2)]/7. This created a continuous variable. A binary variable was then coded using 7 h as a cut off, as per the recommended daily sleeping hours by the American Academy of Sleep Medicine [17].

### Potential confounding variables

Age was categorized into five groups: 16 to 29 years, 30 to 34 years, 35 to 49 years, 50 to 64 years, and 65 + years. Gender was a binary variable as males and females. Race was categorized into non-Hispanic white, non-Hispanic black, Hispanic, non-Hispanic Asian, and other. Education level variable was categorized as < high school, high school graduate/GED, and some college or more. Level of income was collected as an income to poverty ratio. Body Mass Index (BMI) was collected and used as a continuous variable. All variables were derived directly from the NHANES dataset.

### Statistical analysis

Chi square statistical analysis was conducted to describe categorical variables as proportions and counts, and t test analysis was used to describe continuous variables as means and standard deviations, stratified by untreated dental caries and dental caries experience.

Weighted multivariate Poisson and Logistic regression analyses were conducted to test the hypothesis that less sleeping duration might be associated with increased dental caries, adjusting for age, gender, race, level of education, income level, obesity, and BMI. The main outcome variables were used as count outcomes in the Poisson regression and as binary outcomes in Logistic regression analysis. The main exposure variable (sleep duration) was used as both continuous and binary variables as mentioned above.

All analyses were performed using STATA 17 software, while considering the significance level of 0.05.

## Results

Among 5,205 participants aged 16 years and above, 26.2% (1362/5205) had at least one tooth with untreated dental caries. Overall, 31.8% (371/1167) of the participants who slept fewer than 7 h had at least one tooth with untreated dental caries, and 24.6% (991/4038) of participants who slept 7 h or more had at least one tooth with untreated dental caries (Table 1).

**Table 1:** Descriptive and demographics of individuals over the age of 16 stratified by untreated dental caries. Data were extracted from NHANES 2017–2018 cycle

*Independent Variable*		*No Untreated Dental Caries* *n/N (%)*	*At least One tooth with Untreated Dental caries* *n/N (%)*	*P Value*
		**3843/5205 (73.8)**	**1362/5205 (26.2)**	
**Sleep Duration**	**< 7 h**	**796/1167 (68.2)**	**371/1167 (31.8)**	**P < 0.001**
	≥ **7 h**	**3047/4038 (75.6)**	**991/4038 (24.6)**	
**Age**	**16–29**	**1034/1297 (79.7)**	**263/1297 (20.3)**	**P < 0.001**
	**30–34**	**281/405 (69.4)**	**124/405 (30.6)**	
	**35–49**	**792/1104 (71.7)**	**312/1104 (28.3)**	
	**50–64**	**955/1334 (71.6)**	**379/1334 (28.4)**	
	**≥ 65**	**781/1065 (73.3)**	**284/1065 (26.7)**	
**Gender**	**Males**	**1775/2506 (70.8)**	**731/2506 (29.2)**	**P < 0.001**
	**Females**	**2068/2699 (76.6)**	**631/2699 (23.4)**	
**Race/ethnicity**	**Non-Hispanic White**	**1305/1732 (75.4)**	**427/1732 (24.6)**	**P < 0.001**
	**Non-Hispanic Black**	**752/1202 (62.6)**	**450/1202 (37.4)**	
	**Hispanic**	**933/1224 (76.2)**	**291/1224 (23.8)**	
	**Non-Hispanic Asian**	**651/770 (84.6)**	**119/770 (15.4)**	
	**Other race**	**202/277 (72.9)**	**75/277 (27.1)**	
**Education level**	**< High school**	**540/856 (63.1)**	**316/856 (36.9)**	**P < 0.001**
	**High school graduate / GED**	**681/1067 (63.8)**	**386/1067 (36.2)**	
	**Some college or more**	**2131/2723 (78.3)**	**592/2723 (21.7)**	
**Income (Income: Poverty)**	**< 1.38**	**873/1381 (63.2)**	**508/1381 (36.8)**	**P < 0.001**
	**1.38–3.99**	**1427/1952 (73.1)**	**525/1952 (26.9)**	
	**> 3.99**	**1543/1872 (82.4)**	**329/1872 (17.6)**	
**Obesity by BMI**	**Obese < 30**	**2352/3074 (76.5)**	**722/3074 (23.5)**	**P < 0.001**
	**Obese** ≥ **30**	**1491/2131 (70)**	**640/2131 (30)**	
**Average sleep duration**		**Mean = 7.9** **(SD = 1.4)**	**Mean = 7.7** **(SD = 1.6)**	**P < 0.001**
**BMI**		**Mean = 29.1** **(SD = 7.2)**	**Mean = 30.4** **(SD = 8.2)**	**P < 0.001**

P values were determined using chi-square and t-test

Income: Ratio of Family income to poverty

Obesity: considered obese if the BMI was ≥ 30

Body Mass Index: body mass divided by the square of the body height and is expressed in units of kg/m² as a continuous variable

Average sleep duration: number of sleep hours/night on both weekend and weekdays

Our analyses showed that 88.9% (4630/5205) of the study population had experienced dental caries, and 92.5% (1080/1167) of people who reported sleeping < 7 h had experienced dental caries, as opposed to 87.9% (3550/4038) who slept ≥ 7 h (Table 2).

**Table 2:** Descriptive and demographics of individuals over the age of 16 stratified by dental caries experience. Data were extracted from NHANES 2017–2018 cycle

*Independent Variable*		No Dental Caries Experience n/N (%)	Dental Caries Experience n/N (%)	*P value*
		**575/5205 (11.1)**	**4630/5205 (88.9)**	
**Sleep Duration**	**< 7 h**	**87/1167 (7.5)**	**1080/1167 (92.5)**	**P < 0.001**
	≥ **7 h**	**488/4038 (12.1)**	**3550/4038 (87.9)**	
**Age**	**16–29**	**378/1297 (29.1)**	**919/1297 (70.9)**	**P < 0.001**
	**30–34**	**74/405 (18.3)**	**331/405 (81.7)**	
	**35–49**	**86/1104 (7.8)**	**1018/1104 (92.2)**	
	**50–64**	**33/1334 (2.5)**	**1301/1334 (97.5)**	
	**≥ 65**	**4/1061 (0.4)**	**1061/1065 (99.6)**	
**Gender**	**Males**	**297/2506 (11.8)**	**2209/2506 (88.2)**	**P = 0.074**
	**Females**	**278/2699 (10.3)**	**2421/2699 (89.7)**	
**Race/ethnicity**	**Non-Hispanic White**	**167/1732 (9.6)**	**1565/1732 (90.4)**	**P < 0.001**
	**Non-Hispanic Black**	**113/1202 (9.4)**	**1089/1202 (90.6)**	
	**Hispanic**	**128/1224 (10.4)**	**1096/1224 (89.6)**	
	**Non-Hispanic Asian**	**129/770 (16.7)**	**641/770 (83.3)**	
	**Other race**	**38/277 (13.7)**	**239/277 (86.3)**	
**Education level**	**< High school**	**59/856 (6.9)**	**797/856 (93.1)**	**P = 0.28**
	**High school graduate / GED**	**81/1067 (7.6)**	**986/1067 (92.4)**	
	**Some college or more**	**231/2723 (8.5)**	**2492/2723 (91.5)**	
**Income (Income: Poverty)**	**< 1.38**	**151/1381 (10.9)**	**1230/1381 (89.1)**	**P = 0.434**
	**1.38–3.99**	**204/1952 (10.4)**	**1748/1952 (89.6)**	
	**> 3.99**	**220/1872 (11.7)**	**1652/1872 (88.3)**	
**Obesity by BMI**	**Obese (BMI < 30)**	**391/3074 (12.7)**	**2683/3074 (87.3)**	**P < 0.05**
	**Obese (BMI** ≥ **30)**	**184/2131 (8.6)**	**1947/2131 (91.4)**	
**Average sleep duration**		**Mean = 8.1** **(SD = 1.2)**	**Mean = 27.7** **(SD = 7.6)**	**P < 0.05**
**BMI**		**Mean = 7.8** **(SD = 1.5)**	**Mean = 29.7** **(SD = 7.4)**	**P < 0.05**

P values were determined using chi-square and t-test

Income: Ratio of Family income to poverty

Obesity: considered obese if the BMI was ≥ 30

Average sleep duration: number of sleep hours/night on both weekend and weekdays

Body Mass Index: body mass divided by the square of the body height and is expressed in units of kg/m² as a continuous variable

Furthermore, dental caries was evident in all age groups; however, the highest percentage of having at least one tooth with untreated dental caries 30.6% (124/405) was observed in individuals aged between 30 and 34 (Table 1). Additionally, all age groups experienced dental caries, however people aged ≥ 65 presented with the highest percentage of dental caries experience (99.6%) (1061/1065); contrary to individuals aged between 16 and 29 who exhibited the lowest percentage of dental caries experience, at 70.9% (919/1297) (Table 2).

Males exhibited higher levels of at least one tooth with untreated dental caries (29.2%) (731/2506) when compared to females (23.4%) (631/2699) (Table 1).

For a one hour increase in average sleep hours, the Adjusted Mean Ratio (AMR) of untreated dental caries might decrease by 0.89 (AMR = 0.89, 95% CI = 0.81–0.98, P < 0.05). The mean number of untreated dental caries was lower for those who reported sleeping ≥ 7 h/night than individuals who reported sleeping < 7 h/night (AMR = 0.69, 95% CI = 0.57–0.83, P < 0.05). For a one hour increase in average sleep hours, the AMR of dental caries experience might decrease by 0.97 (AMR = 0.97, 95% CI = 0.96–0.99, P < 0.05). The mean number of dental caries experience was lower for those who reported sleeping ≥ 7 h/night than individuals who reported sleeping < 7 h/night (AMR = 0.92, 95% CI = 0.87–0.99, P < 0.05) (Table 3).

**Table 3:** Weighted multivariate Poisson regression analyses of the association between untreated dental caries and dental caries experience with sleep for individuals aged 16 or above

Predictor variable	Untreated Dental Caries			Dental Caries Experience		
	Adjusted Mean Ratio	P Value	95% Confidence Interval	Adjusted Mean Ratio	P Value	95% Confidence Interval
Sleep duration*	0.89	P = 0.023	0.81–0.98	0.97	0.004	0.96–0.99
Sleep ≥ 7 h/night#	0.69	P = 0.001	0.57–0.83	0.92	0.03	0.87–0.99

All models were adjusted for age, gender, race, level of education, income level and BMI

*: Sleep duration is a continuous variable for the number of hours slept per night

#: A binary variable for those who slept ≥ 7 h per night vs. those who slept < 7 h

For a one hour increase in average sleep hours, the Adjusted Odds Ratio (AOR) of untreated dental caries might decrease by 0.87 (AOR = 0.87, 95% CI = 0.81–0.94, P < 0.05). Individuals who reported an average sleep of ≥ 7 h were less likely to have untreated dental caries compared to individuals who reported an average sleep of < 7 h (AOR = 0.7, 95% CI = 0.56–0.88, P < 0.05). For a one hour increase in average sleep hours, the AOR of experiencing dental caries might decrease by 0.86 (AOR = 0.86, 95% CI = 0.75–0.98, P < 0.05). Individuals who reported an average sleep of ≥ 7 h were less likely to experience dental caries compared to individuals who reported an average sleep of < 7 h (AOR = 0.52, 95% CI = 0.33–0.82, P < 0.05) (Table 4).

**Table 4:** Weighted multivariate Logistic regression analyses of the association between untreated dental caries and dental caries experience with sleep for individuals aged 16 or above

Predictor variable	Untreated Dental Caries			Dental Caries Experience		
	Adjusted Odds Ratio	P Value	95% Confidence Interval	Adjusted Odds Ratio	P Value	95% Confidence Interval
Sleep duration*	0.87	0.002	0.81–0.94	0.86	0.036	0.75–0.98
Sleep ≥ 7 h/night#	0.7	0.005	0.56–0.88	0.52	0.008	0.33–0.82

All models were adjusted for age, gender, race, level of education, income level and BMI

*: Sleep duration is a continuous variable for the number of hours slept per night

#: A binary variable for those who slept ≥ 7 h per night vs. those who slept < 7 h

## Discussion

This study has demonstrated that decreased sleep duration was associated with higher levels of dental caries. There is scant evidence in the literature on the relationship between dental caries and short sleep duration in adults. However, this result is consistent with a cohort study conducted among 5,456 Kuwaiti children showing that children who go to bed late have increased dental caries incidence compared to those who go to bed early [18]. Similarly, a study by Asaka et al. showed that children who sleep less than eight hours a day were 1.5 times more likely to have tooth decay [15]. Another cohort study conducted in Japan showed that late bedtime and short sleep duration were consistently correlated with an increased risk of caries in deciduous teeth [14].

There is established evidence that sleep behavior can control appetite [19]. Studies have shown that decreased sleep duration is associated with increased levels of ghrelin, a hormone which increases appetite, and a decrease in leptin, a hormone which decreases appetite [20]. A study on sleep curtailment found those with shortened sleep duration were more likely to have an increased caloric intake by snacking more frequently, thus contributing to the development of dental caries [21]. It was also reported that an increase in food intake in people who sleep less can be explained through a change in the cognitive function that affects the inhibition and reward system [22], or due to an increase in opportunities for snacking during the late night wake time [23].

A strong body of evidence has linked short sleep duration with higher BMI and obesity [24–26]. Importantly, a study by Arvidsson et al., found that higher BMI score was positively associated with higher counts of cariogenic bacteria Mutans Streptococci, further contributing to the risk of the development of dental caries [27]. Although multiple studies have reported a positive association between obesity and dental caries, there is inconclusive evidence in the literature on this association among children and adults [28–30]. In our study, after adjusting for BMI as a potential confounder, we observed a negative association between dental caries and sleep duration.

In addition, evidence suggested that insufficient sleep can reduce the salivary flow rate and eventually promote dental caries [31]. The flow of saliva is regulated by the circadian rhythm, which is controlled by healthy sleep behavior [32]. It is well known that salivary flow plays an important role in the bacterial clearance, dilution, and buffering abilities of saliva [33]. An optimum salivary flow rate is crucial for establishing a protective environment against dental caries [32–34]. Consequently, a reduction in salivary flow rate implies a drastic alteration in the oral clearing and buffering capacity which may explain why sleep deprivation is associated with a higher incidence of dental caries [32–34]. It was indicated that the risk of increasing dental caries in night shift workers is linked to the reduction in salivary flow rate [35]. Nevertheless, the mechanisms by which sleep affects salivary flow are not yet fully understood, but it is thought that the release of certain hormones, such as cortisol, may play a role [34].

Furthermore, research has found that the levels of salivary amylase are affected by the sleep-wake cycle [36], and that salivary amylase activity was significantly increased in the sleep deprived group compared to the control group [31]. It is worth mentioning that salivary amylase has an important role on dental plaque and caries development [35]. Salivary amylase breaks down carbohydrates, which can then be converted into sugar by bacteria in the mouth, leading to the production of acid that erodes enamel and contributes to the formation of dental caries.

Lasisi et al. reported that the secretion rate of salivary Immunoglobulin A (IgA) was significantly decreased in the sleep deprived group in comparison to a control group [31]. Salivary IgA (s-IgA) is the most abundant antibody and has a crucial role in preventing colonization by pathogens as it is considered the first line of defense against pathogens that enter the body through the mouth and respiratory tract [31]. It is also suggested that s-IgA can be a predictor of caries development [32]. Nevertheless, another study showed conflicted associations between s-IgA and dental caries [37].

Roestamadji et al. found that individuals who work night shifts and experience circadian misalignment have higher fasting blood glucose (FBG) levels, which has been positively correlated with an increased DMFT (Decayed, Missing, Filled, Teeth) index [35].

A study by Bryant et al. indicated that a disrupted circadian rhythm can lead to a diminished immune response [8]. It has been observed that alterations in cortisol, and melatonin, which are regulated by sleep, frequently have an effect on immune functions [9]. A diminished immune system may allow for the flourishing of oral bacteria and consequent susceptibility to dental caries. It has been discovered that in hamsters, a species known for its seasonal behavior, there was an increase in the number of carious lesions during the spring and summer, when the duration of nocturnal elevated melatonin is at its lowest [38]. Conversely, caries was less common during the autumn and winter, when melatonin levels were at their highest [39]. A study revealed that individuals suffering from dental caries with a DMFT score exceeding 10 had considerably lower levels of melatonin in their saliva in comparison to those without dental caries [39]. Melatonin has been investigated as a potential countermeasure against the oxidative stress that is induced during the onset and progression of dental caries [40].

One limitation of this study is the utilization of self-reported sleep behavior which can be subjected to reporting and recall bias. This paper is a cross-sectional study, limiting the ability to assess temporality, and so, causality cannot be measured. Furthermore, the NHANES does not collect data from institutionalized individuals, which limits our ability to evaluate the association between sleep duration and dental caries in vulnerable institutionalized populations. Additionally, factors that play a significant role in the development of dental caries such as sugar intake, frequency of oral hygiene measures and use of Fluoridated toothpaste were not reported in the study population. It is important to note that our study was limited to two years of NHANES data (2017-2018), which may provide unstable estimates for some populations and age groups. On the other hand, this study has some strengths, NHANES data is a nationally representative sample, so selection bias was minimized. Although we controlled for known confounding factors to strengthen the internal validity of the estimates, there might be unmeasured potential confounders for developing dental caries such as specification and timing of snacking.

## Conclusion

Findings of this cross-sectional representative study of the U.S. population revealed a statistically significant negative relationship between sleep duration and dental caries. In this study, individuals who slept ≥ 7 h/night were less likely to exhibit dental caries compared to those who slept < 7 h. Additional longitudinal studies are necessary to elucidate the underlying biological mechanisms by which insufficient sleep duration affects various oral parameters that play a role in the development of dental caries. These parameters include the oral microbiome, pH level, and other oral biomarkers.

## Data Availability

The datasets generated and/or analyzed during the current study are available in the National Health and Nutrition Examination Surveys (NHANES) repository, [https://wwwn.cdc.gov/nchs/nhanes/continuousnhanes/default.aspx?BeginYear=2017]. Accessed January 30, 2023.

## Abbreviations

NHANES: National Health and Nutrition Examination Survey NHANES
CDC: Center for Disease Control and Prevention
US: United States
DMFT Index: D:Decayed, M:Missing, F:filled
BMI: Body Mass Index
OR: Odds Ratio
AMR: Adjusted Mean Ratio
IgA: Immunoglobulin A
s-IgA: Salivary Immunoglobulin A
FBG: Fasting Blood Glucose
